# Comparative genomics of 40 *Weissella paramesenteroides* strains

**DOI:** 10.3389/fmicb.2023.1128028

**Published:** 2023-03-31

**Authors:** Xing Wan, Timo M. Takala, Vy A. Huynh, Susanna L. Ahonen, Lars Paulin, Johanna Björkroth, Tarja Sironen, Ravi Kant, Per Saris

**Affiliations:** ^1^Department of Microbiology, Faculty of Agriculture and Forestry, University of Helsinki, Helsinki, Finland; ^2^Department of Bacteriology and Immunology, Human Microbiome Research Program, Faculty of Medicine, University of Helsinki, Helsinki, Finland; ^3^Finnish Institute for Health and Welfare, Helsinki, Finland; ^4^Institute of Biotechnology, University of Helsinki, Helsinki, Finland; ^5^Department of Food Hygiene and Environmental Health, Faculty of Veterinary Medicine, University of Helsinki, Helsinki, Finland; ^6^Department of Virology, Faculty of Medicine, University of Helsinki, Helsinki, Finland; ^7^Department of Veterinary Biosciences, Faculty of Veterinary Medicine, University of Helsinki, Helsinki, Finland; ^8^Department of Tropical Parasitology, Institute of Maritime and Tropical Medicine, Medical University of Gdańsk, Gdynia, Poland

**Keywords:** *Weissella paramesenteroides*, comparative genomics, pangenome analysis, core genome, vancomycin resistance, heterofermentative bacteria

## Abstract

*Weissella* strains are often detected in spontaneously fermented foods. Because of their abilities to produce lactic acid and functional exopolysaccharides as well as their probiotic traits, *Weissella* spp. improve not only the sensorial properties but also nutritional values of the fermented food products. However, some *Weissella* species have been associated with human and animal diseases. In the era of vast genomic sequencing, new genomic/genome data are becoming available to the public on daily pace. Detailed genomic analyses are due to provide a full understanding of individual *Weissella* species. In this study, the genomes of six *Weissella paramesenteroides* strains were *de novo* sequenced. The genomes of 42 *W. paramesenteroides* strains were compared to discover their metabolic and functional potentials in food fermentation. Comparative genomics and metabolic pathway reconstructions revealed that *W. paramesenteroides* is a compact group of heterofermentative bacteria with good capacity of producing secondary metabolites and vitamin Bs. Since the strains rarely harbored plasmid DNA, they did not commonly possess the genes associated with bacteriocin production. All 42 strains were shown to bear *vanT* gene from the glycopeptide resistance gene cluster vanG. Yet none of the strains carried virulence genes.

## Introduction

*Weissella* species are Gram-positive coccoid or rod-shaped lactic acid bacteria (LAB) (Fusco et al., 2015), which have been separated from the genus *Leuconostoc* (Collins et al., 1993). *Weissella* species are catalase-negative and intrinsically vancomycin-resistant. They have received considerable interests in the food industry due to their probiotic characters, the ability of converting carbohydrates into lactic acids, and the production of functional exopolysaccharides (Kavitake et al., 2020). Although *Weissella* spp. are not regarded as GRAS (Generally Recognized as Safe), they have great potential to be used as starters in food fermentation along with *Lactococcus* and *Leuconostoc* (Fessard and Remize, 2017). So far, 19 *Weissella* species have been described including *W. beninensis*, *W. ceti*, *W. cibaria*, *W. confusa*, *W. diestrammenae*, *W. fabalis*, *W. fabaria*, *W. ghanensis*, *W. halotolerans*, *W. hellenica*, *W. kandleri*, *W. koreensis*, *W. minor*, *W. oryzae*, *W. paramesenteroides*, *W. soli*, *W. thailandensis*, *W. uvarum*, and *W. viridescens* (Fusco et al., 2015). Among those species, *W. cibaria*, *W. confusa* strains, and *W. viridescens* are often associated with clinical infections in human (Fusco et al., 2015).

*Weissella paramesenteroides* received species status first under the name *Leuconostoc paramesenteroides* in 1967, based on the difference in the utilization of amino acids and vitamins requirements and the failure on esculin and salicin hydrolyzation; it was distinguished from *Leuconostoc mesenteroides* (Garvie, 1967). Only until 1993, when Collins et al. (1993) performed taxonomic studies using comparative 16S rRNA sequence analysis, *L. paramesenteroides* was proposed to be reclassified in a new genus *Weissella*.

*Weissella* species can be found from different vegetable, meat, and fish fermentations (Fessard and Remize, 2017), as well as various traditional fermented foods including Indian idli and dahi (Patel et al., 2013; Sharma et al., 2018). In fermentations, *Weissella* spp. contributes to the production of exopolysaccharides (Patel et al., 2012). Lactic acid bacteria (LAB) and yeasts play a predominant role in the natural fermentation of idli batter. These organisms originate during soaking of rice and black gram aiding spontaneous fermentation accompanied by two notable changes in idli batter—acidification and leavening (Mukherjee et al., 1965). *W. paramesenteroides*, in particular, has caught attention on its potential of producing antimicrobial compounds and exopolysaccharides, as well as its probiotic and prebiotic properties (Pabari et al., 2020; Yadav et al., 2022). For example, the probiotic *W. paramesenteroides* WpK4 was shown to reduce both intestinal permeability in colitis mice and anxiety-like behaviors in chronically stressed mice (Sandes et al., 2020). A few bacteriocins have been characterized from *W. paramesenteroides* strains, such as the *Listeria*-inhibiting pediocin-like class IIa bacteriocin weissellin A (Papagianni and Papamichael, 2011).

With the advancing of sequencing techniques, the genomes of various lactic acid bacterial genera have been sequenced. This expansion of genome visibility facilitates the research on comparative genomics, which can not only reveal the full potential of the species as whole but also unfolding the genomic diversity of the dataset. Nevertheless, although 36 genomes of *W. paramesenteroides* are already publicly available in NCBI database by August 2022, a systematic investigation in these genomes was missing. Comparative genomic study would provide a deeper understanding of the species and contribute to the application of probiotic *W. paramesenteroides* strains.

In this study, we isolated 6 *W. paramesenteroides* strains from different food sources and performed whole-genome sequencing on their genomic DNA. We obtained draft genomes of five strains and one complete genomic sequence of the strain W31. From the comparative genomic analysis together with the existing *W. paramesenteroides* genomes, we gained an insight into the genetic characteristics of the *W. paramesenteroides* species and uncovered the diversity and similarity among these publicly available 42 strains. We also investigated the abilities of producing bacteriocins and exhibiting antibiotic resistance of these *W. paramesenteroides*. Their metabolic capabilities were also studied in detail, to enable better use of these strains in food fermentation.

## Materials and methods

### Bacterial isolates, growth conditions, DNA extraction, and antimicrobial assay

Idli batter was prepared from parboiled rice (*Oryza sativa*) and dehulled black gram (*Phaseolus mungo*) as described as before (Chandrasekar Rajendran et al., 2017). *W. paramesenteroides* strains W31, B1, and C8 were isolated from the overnight fermented batter on De Man, Rogosa and Sharpe (MRS) agar plates supplemented with 1% sucrose using dilutions and plating methods (Sanders, 2012). Strains SJ21-9, SJ27-4, and SJ27-5 were isolated from air dried onion powders (Säde et al., 2016). All isolates were grown in MRS liquid media supplemented with 1% sucrose.

For genome sequencing, total bacterial DNAs from 2 ml cultures of *W. paramesenteroides* isolates grown overnight in MRS at 30°C were extracted using MagAttract HMW DNA kit (Qiagen, Hilden, Germany) according to the supplier’s protocol.

Conventional spot-on-lawn method was used to test the antimicrobial activity of *W. paramesenteroides* strains. The indicator strain *Micrococcus luteus* NCIMB 86166 was grown in LB broth at 37°C overnight. Four hundred microliters of the indicator culture was mixed with 1 ml of sterile water, poured onto a LB agar plate and let dried. Ten μl of *Weissella* overnight cultures were spotted onto the surface of the *M. luteus* indicator plate, and the plate was incubated overnight at 28°C.

### Genome sequencing, assembly, and annotation

Genomes of the six *Weissella paramesenteroides* isolates were sequenced at the Institute of Biotechnology (University of Helsinki, Finland) using next-generation sequencing platforms. Genomic DNA (0.5 mg) was sheared using a Bioruptor NGS Sonicator (Diagenode) to approximately 600 bp fragments. The fragments were polished, A-tailed, and ligated to a TruSeq truncated adapter. Purification of the ligation reaction was done using AMPure XP beads (Agencourt, Beckman Coulter). PCR of the libraries were done using Phusion Hot Start II DNA Polymerase (Thermo Fisher Scientific, Waltham, MA, USA) and index P7 primers and full-length P5 adapter primers. The reactions were pooled and purified with AMPure XP beads. Size selection of the pool was done according to Lundin et al. (2010). The obtained library pool was paired-end sequenced on a MiSeq Sequencer using the v3 600 cycle kit (Illumina, San Diego, CA, USA).

The genomes were assembled using the SPAdes assembly pipeline v3.15.5 with default setting (Prjibelski et al., 2020). Plasmids were identified using the plasmid option of SPAdes (Antipov et al., 2016). The quality of the assemblies was determined with Quast (v5.2.0, Mikheenko et al., 2018) using default settings and the genome of *W. paramesenteroides* STCH-BD1 as reference. Contigs with length shorter than 500 bp were discarded.

Genomes of the six newly sequenced *W. paramesenteroides* strains were deposited in GenBank under the accession numbers listed in Table 1. The annotation was performed using the assembled DNA sequences of the six new draft genomes from these isolates. The genomes were run through an automatic annotation pipeline PGAP (NCBI Prokaryotic Genome Annotation Pipeline) (Tatusova et al., 2016), followed by manual curation in few cases.

**TABLE 1:** A general overview of 42 *Weissella paramesenteroides* genomes.

Strain	Source	BioSample	Status	Coverage	Contigs	Size (Mbps)	G + C (%)	ORFs	Proteins	References
WP-W31	Idli batter	SAMN14332707	Complete	100	2	2.03	38.2	2026	1893	This study
WP-B1	Idli batter	SAMN14332709	Draft	90	12	2	37.9	2005	1891	This study
WP-C8	Idli batter	SAMN14332710	Draft	120	19	1.92	37.9	1940	1830	This study
WP-SJ21-9	Dried onion powder	SAMN14332723	Draft	75	35	2.1	37.9	2164	2041	This study
WP-SJ27-4	Dried onion powder	SAMN14332761	Draft	130	36	2.1	37.9	2170	2042	This study
WP-SJ27-5	Dried onion powder	SAMN14332762	Draft	103	35	2.1	37.9	2142	2016	This study
3.2.24	NA	SAMN18995137	Draft	238	31	1.95	38	1936	1833	DS
A37	*Meles meles*	SAMN10822536	Draft	20	20	1.94	37.9	1955	1826	Stedman et al., 2018
ATCC 33313	*Homo sapiens*	SAMN00139194	Draft	NA	13	1.98	38	1978	1873	DS
DmW_107	*Drosophila melanogaster*	SAMN12113445	Draft	89	9	2.02	38	2043	1935	Rudman et al., 2019
DmW_107099	*Drosophila melanogaster*	SAMN12113442	Draft	155	48	1.99	37.9	2009	1911	“
DmW_107100	*Drosophila melanogaster*	SAMN12113443	Draft	42	47	1.99	37.9	2004	1911	“
DmW_107101	*Drosophila melanogaster*	SAMN12113447	Draft	263	48	1.99	37.9	2009	1912	“
DmW_107102	*Drosophila melanogaster*	SAMN12113446	Draft	51	50	1.99	37.9	2010	1913	“
DmW_107104	*Drosophila melanogaster*	SAMN12113448	Draft	255	49	1.99	37.9	2013	1917	“
DmW_107105	*Drosophila melanogaster*	SAMN12113449	Draft	139	47	1.99	37.9	2011	1919	“
DmW_107106	*Drosophila melanogaster*	SAMN12113444	Draft	186	48	1.96	37.9	1986	1890	“
DmW_107108	*Drosophila melanogaster*	SAMN12113450	Draft	57	26	1.97	37.9	1969	1878	“
DmW_109	*Drosophila melanogaster*	SAMN12113452	Draft	100	21	1.93	37.9	1934	1842	“
DmW_109110	*Drosophila melanogaster*	SAMN12113451	Draft	142	22	1.82	38	1828	1741	“
DmW_115	*Drosophila melanogaster*	SAMN12113454	Draft	124	27	2.00	37.9	2005	1907	“
DmW_115112	*Drosophila melanogaster*	SAMN12113455	Draft	47	28	2.00	37.9	2006	1910	“
DmW_115113	*Drosophila melanogaster*	SAMN12113456	Draft	153	24	2.00	37.9	2002	1906	“
DmW_115114	*Drosophila melanogaster*	SAMN12113457	Draft	45	25	2.00	37.9	1998	1905	“
DmW_115116	*Drosophila melanogaster*	SAMN12113453	Draft	374	24	2.00	37.9	2003	1906	“
DmW_118*	*Drosophila melanogaster*	SAMN12113459	Draft	77	46	1.81	36.5	1774	1680	“
DmW_118119*	*Drosophila melanogaster*	SAMN12113458	Draft	97	49	1.81	36.5	1772	1676	“
DRD-105	Artisanal Greek cheeses	SAMN28910668	Draft	371	27	1.95	37.9	1986	1891	DS
DRD-137	Artisanal Greek cheeses	SAMN28910669	Draft	407	25	1.95	37.9	1985	1890	DS
DRD-142	Artisanal Greek cheeses	SAMN28910670	Draft	358	7	1.75	38.3	1736	1601	DS
DRD-149	Artisanal Greek cheeses	SAMN28910671	Draft	485	25	1.95	37.9	1987	1892	DS
DRD-169	Artisanal Greek cheeses	SAMN28910672	Draft	400	26	1.91	38	1945	1850	DS
DRD-172	Artisanal Greek cheeses	SAMN28910673	Draft	341	21	1.91	38	1945	1851	DS
DRD-187	Artisanal Greek cheeses	SAMN28910674	Draft	351	24	1.95	37.9	1986	1891	DS
DRD-194	Artisanal Greek cheeses	SAMN28910675	Draft	344	26	1.95	37.9	1986	1889	DS
DRD-39	Artisanal Greek cheeses	SAMN28910667	Draft	354	25	1.95	37.9	1982	1890	DS
FDAARGOS _414	Environment	SAMN07312458	Complete	2770	1	1.95	38.1	1923	1781	DS
MGYG-HGUT-01340	NA	SAMEA5850843	Draft	10	13	1.98	38	1977	1872	DS
N43	*Meles meles*	SAMN10822537	Draft	20	20	1.94	37.9	1956	1826	Stedman et al., 2018
STCH-BD1	Ensiled *Sorghum bicolor*	SAMN16830909	Complete	210	2	2.05	38.1422	2057	1924	DS
TR212	Sourdough	SAMN09099489	Draft	137	22	1.98	38.1	2011	1876	DS
WpK4	Piglets	SAMN10880043	Draft	80	10	2.04	38.2924	2016	1866	DS

NA, not applicable; DS, sequences were direct submission to NCBI without a published article; “, same as previous row.

*Species outliers.

### Pan-genome comparison and phylogenetic analysis

To find out the phylogenetic relation between our isolates and other *W. paramesenteroides* strains, we reconstructed the phylogeny using all available 34 *W. paramesenteroides* genomes retrieved from NCBI genome (dataset downloaded by latest 6th July 2022, Table 1) together with the six newly constructed genomes. The pangenomes of all 42 *W. paramesenteroides* strains were analyzed using Roary pipeline version 3.13 (Page et al., 2015). First, the nucleotide fasta files and GFF files from the published 34 strains were downloaded from NCBI and converted to GFF3 using a script (Supplementary Datasheet 1). For strains which GFF files were missing in NCBI or could not pass the preliminary analysis in Roary pipeline, their nucleotide fasta files were annotated using Prokka (v1.14.6) to obtain a compliable GFF3 file (Seemann, 2014). The 42 GFF3 annotations were then fed to Roary to calculate the pangenome of the dataset and produce a multiple sequence alignment (MSA) of the core genes (present in > 99% strains) using MAFFT. The obtained MSA of the core genome was used to generate a best-fit maximum likelihood phylogeny using IQTREE version 1.6.12 using ModelFinder optimization (Kalyaanamoorthy et al., 2017). Finally, the trees were visualized in an R package ggtree (Yu, 2020). A python script roary_plots.py^1^ was used to show the presence and absence of core and accessory genes. All genomic fasta files were quality controlled to eliminate shorter sequences under the length of 300 nucleotide prior to Average Nucleotide Identity (ANI) analysis. The ANI of all genomes was calculated using a python module pyani v0.2.7 (Pritchard et al., 2016).

### Functional prediction

To predict the presence of bacteriocin encoding genes, 42 *W. paramesenteroides* genomes were analyzed by online prediction tool BAGEL4.^2^ Any resultant genomic area of interest from BAGEL4 was analyzed further using BLASTP (Altschul et al., 1997). Virulence and the presence of any antibiotic resistance genes were predicted by VirulenceFinder 2.0^3^ and in RGI tool,^4^ respectively. Prophage sequences were searched using PHAST (Zhou et al., 2011), while the CRISPRs and Cas genes developed by bacteria for phage defensing were searched on CRISPRCasFinder (Couvin et al., 2018). To further understand the function of genomes even with novel sequences, the core genome of the *W. paramesenteroides* was annotated through eggNOG-mapper v2.^5^ The metabolic pathways were reconstructed using KEGG mapper.^6^

## Results

### General features of the *W. paramesenteroides* genomes

In this study, we sequenced the genomes of six *W. paramesenteroides* isolates. The genome of strain W31 was complete, while the other five draft genomes contain 2 to 36 contigs. For all the assemblies, 1−3 contigs together constituted more than 50% of the genome size. The six newly assembled genomes had a mean size of 2.035 Mbp, from 1.92 Mbp of strain C8 to 2.1 Mbp of the three SJ strains. The G + C content of these strains was 37.9%, except *W. paramesenteroides* W31, which contains higher GC percentage of 38.2% (Table 1). An over 75-fold coverage was obtained for the six newly sequenced genomes. One plasmid was identified from *W. paramesenteroides* strains B1 and W31 each, and strain C8 seemed to carry three plasmids. Since the genome assemblies of the three SJ strains contained more fragmented contigs, plasmidSPAdes could only detect short contigs from their genomes as putative plasmids, while the plasmid status of these detected contigs were still questionable (Supplementary Table 1). To confirm the existence of extrachromosomal DNA of these strains, plasmid isolation, and sequencing would be needed. Closing gaps between contigs e.g., using hybrid assembly would also provide a better view on the plasmids of these newly assembled genomes. Having plasmid DNA is an uncommon feature of *W. paramesenteroides*, as among the previously published 34 *W. paramesenteroides* genomes only *W. paramesenteroides* STCH-BD1 and WpK4 contain plasmids with the latter having extraordinary seven plasmids. The genomes of two strains DmW_118119 and DmW_118 were excluded from several further analyses, due to their failure in taxonomy check by NCBI and share less than 50% symmetric identities with the other *W. paramesenteroides* strains.

**FIGURE 1:**
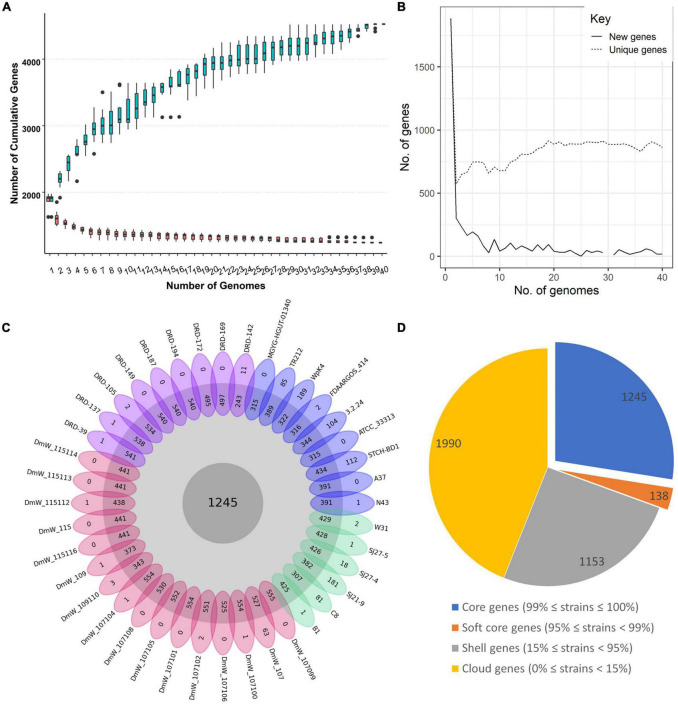
Core genome and pan genome of the 40 *Weissella paramesenteroides* strains. **(A)** The Pan-genome development of *W. paramesenteroides* by including the genomes one-by-one. **(B)** The number of unique and new genes in the pangenome. **(C)** A flower plot indicating the core genes in the center, the number of accessory genes for each strain in the outer circle, and unique genes for each strain in the petals, DRD strains in purple, DmW strains in red, our strains in green, individual strains in blue. **(D)** The summary of CDS distribution among the strains presented in a pie chart, core genes- present in all of the strains, soft core genes- present in more than 38 of the strains, shell genes- present in 6–37 of the strains, cloud genes- present in one or two strains only.

The six new genomes contain coding sequences (CDSs) ranging between 3389 and 3469, with an average of 3,407 genes. To identify the orthologous genes, all the CDSs were compared by blastp by all-against-all strategy. The pangenome of *W. paramesenteroides* without the outliers DmW_118 and DmW_118119 included 4,526 orthologous genes and the core genome contained 1,245 genes in addition to the softcore genome (138 genes) which together take the share of 30.56% of the total genes. The accessory genome includes 1,153 shell genes and 1,990 cloud genes (Figure 1D). The cumulative gene curve of 40 *W. paramesenteroides* pangenome fit the Heap’s law (*n* = κN^γ^). The value of γ was calculated to be 0.85 (< 1.0) for the 40 genomes, using the result of the present and absent genes from roary pipeline, with the heaps () function in R package micropan (Snipen and Liland, 2015). Therefore, *W. paramesenteroides* pangenome was considered open (Figure 1A; Tettelin et al., 2008). The numbers of accessory genes in individual strains and numbers of unique genes varied between genomes (Figure 1C), while the number of unique genes stabilized below 900 (Figure 1B).

### Phylogenetic analysis

**FIGURE 2:**
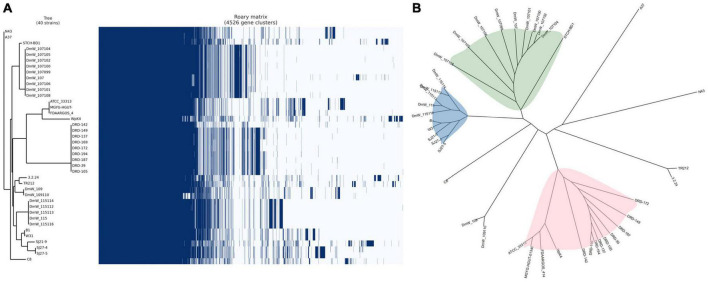
**(A)** Pangenome matrix of the 40 *Weissella paramesenteroides*. Blue, gene presence; white, gene absence. **(B)** Unrooted tree of the 40 *W. paramesenteroides* divided into three major clades.

The roary matrix based on the presence and absence of the genes among 40 *W. paramesenteroides* genomes was generated and is shown in Figure 2A. Two genomes of strains DmW_118119 and DmW_118 were excluded from the matrix here. Nevertheless, we still performed the phylogenetic analysis on all 42 strains (Supplementary Figure 1), in which *W. paramesenteroides* DmW_118 and DmW_118119 apparently stood out from the rest of the *Weissella* strains, indicating their phylogenetic differences from the other strains.

The best assembled genomes of strain W31 in our study is closely related to and share the immediate evolutionary origin with strain WpK4. Two other isolates *W. paramesenteroides* SJ27-4 and SJ27-5 are close to each other, as expected from their origin of isolation, which also is reflected the relatedness of SJ21-9 strain to them.

### COG and metabolic pathways of core genome

**FIGURE 3:**
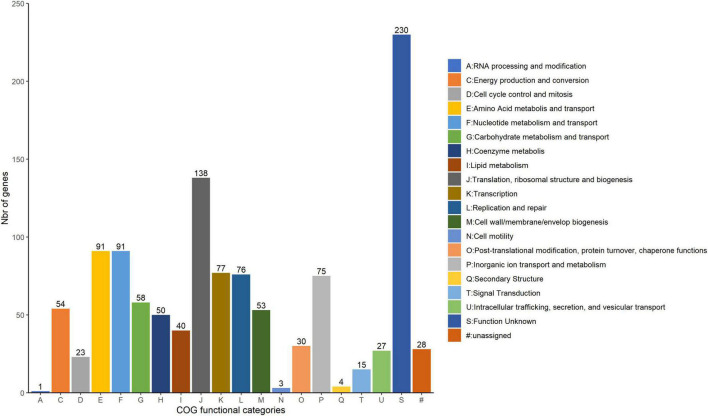
COG category abundances in the core genome of *Weissella paramesenteroides*. The number of genes within each category is indicated above the bar of each category abbreviation.

A comparative analysis of genomic sequences was carried out for the 40 *W. paramesenteroides* genomes excluding DmW_118 and DmW_118119. Of the 1,245 genes of the core genome, 1,164 were successfully scanned by the eggNOG-mapper and ascribed to 20 COG families (Figure 3). Functional annotation of the COGs of the core genome showed that the majority encoded proteins responsible for the information processing systems associated to translation, ribosomal structure, and biogenesis (11.9%), and for amino acid and nucleotide metabolism and transport (7.8% each). The core genome also contained a large number of genes of unknown function (230, 19.8%) and 28 genes which could not be assigned to any functional category in the tested database (2.4%).

The metabolic pathways of the core genome were reconstructed using KEGG gene annotation predicted by eggNOG-mapper. The core genome of *W. paramesenteroides* is heavily involved in biosynthesis of secondary metabolites, cofactors, and amino acids. As shown in Supplementary Table 2, 96 orthologous enzymes are responsible for biosynthesis of secondary metabolites. *W. paramesenteroides* strains utilize 39 core proteins for amino acid biosynthesis, while dedicating an additional 87 orthologous enzymes to amino acid metabolic pathways. Among the 44 essential proteins needed for the synthesis of cofactors, these *Weissella* strains seem to be particularly good at biosynthesis of folate.

### Average nucleotide identity

**FIGURE 4:**
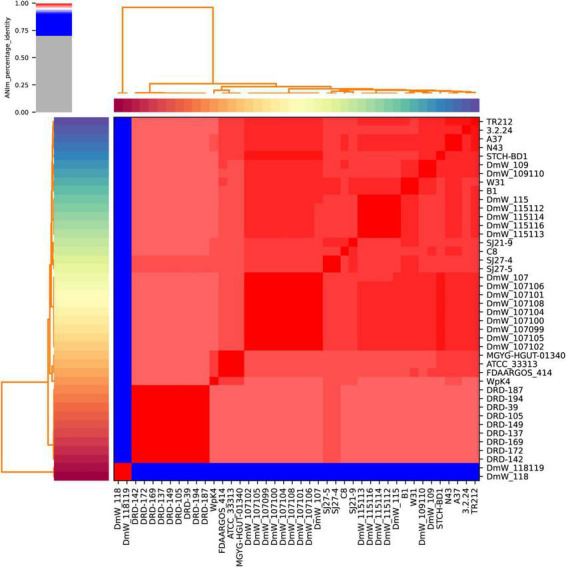
Average Nucleotide Identity among all 42 *Weissella paramesenteroides* strains.

As shown in Figure 4, the pairwise comparison between all the 42 publicly available *W. paramesenteroides* genomes revealed that the 40 true *W. paramesenteroides* genomes had ANI scores 97.7% higher than the threshold for species demarcation (95%, Kim et al., 2014). Two strains DmW_118119 and DmW_118 significantly differ from the other strains, with only 84.4% ANI, as already noticed in the phylogenetic analysis. Interestingly, ANI analysis between strains DmW_118, DmW_118119 and all ten publicly available *Weissella hellenica* genomes could possibly demarcate the two outlier strains to *W. hellenica*, as their ANI were over 99% when compared with six of the *W. hellenica* strains (0916-4-2, 1.2.50, CBA3632, MBEL1842, PC1A, and Wikim14, Supplementary Table 3). Species identification using 16S rRNA sequences also agreed with this observation (data not shown).

### Bacteriophages and phage defense

The presence of prophages in all *W. paramesenteroides* genomes was investigated using search tools PHAST. All strains contained 2−7 phage sequences (Supplementary Table 4). Strains FDAARGOS_414, DRD-142, DmW_118, and DmW_118119 did not carry any intact prophages, but the others contained at least one intact and a few incomplete or questionable prophages per genome. Intact prophage regions spanned from 18.8 kb in strain DRD-187 genome to 57.9 kb in DRD-137 genome. The strain DmW_107 carried extraordinarily seven prophage regions, of which two were intact harboring necessary genes for phage assembly. Strains C8, 3.2.24, A37, N43, and STCH-BD1 carried only intact prophages without any incomplete phage fragments.

Since the prophages were shown to be prevalent among all *W. paramesenteroides* genomes, we wondered whether the phage defensing system CRISPR/Cas was also widespread among those bacteria. Nevertheless, according to the online CRISPRCasFinder tool, none of the 42 *W. paramesenteroides* strains showed strong evidence (evidence level three or four) of the presence of CRISPR sequences and *cas* genes in their genomes (Supplementary Table 5).

### The bacteriocins

**FIGURE 5:**
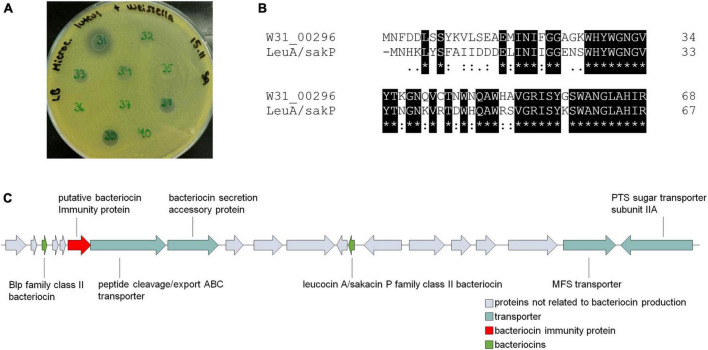
Bacteriocin production potential of *Weissella paramesenteroides.* **(A)** Bactericidal activity of ten *Weissella* idli isolates, showing the inhibitory activity of *W. paramesenteroides* strain W31 against *M. luteus*. Spot number 32 is *W. paramesenteroides* strain B1. **(B)** Amino acid sequence alignment between the gene product Gp00296 from W31 (W31_00296) and the putative leucocin A/sakacin P family class II bacteriocin (LeuA/SakP) identified in DmW_118 and DmW_118119. **(C)** Genomic area including genes for putative bacteriocin production in DmW_118 and DmW_118119.

*Weissella* share similarities with *Leuconostoc* species, which have different roles in foods, for instance as dairy starters, meat spoilage bacteria, and meat protective cultures. The last-mentioned role is based on the production of antimicrobial peptides, bacteriocins. Previously, we had isolated and identified 50 *Weissella* strains from spontaneously fermented idli batter. When testing their antimicrobial potential, we found that *W. paramesenteroides* strain W31 kills other Gram-positive bacteria. As exemplified on *M. luteus* indicator plate with ten *Weissella* idli isolates (Figure 5A), unlike other slightly bactericidal *Weissella* strains 33, 38, and 39, W31 produced larger inhibition zone indicating the production of a bacteriocin (Figure 5A). For this reason, in addition to the strain W31, we screened all 42 *W. paramesenteroides* genomes for the presence of bacteriocin related genes using BAGEL4. The distance of DmW_118 and DmW_118119 from the rest of *Weissella* strains was also shown in the presence of bacteriocin related genes, as based on BAGEL4, only these two strains carried bacteriocin genes. Both strains harbored identical cassette for the production of a leucocin A/sakacin P family class II bacteriocin, including the genes encoding bacteriocin precursors, a putative immunity protein and an ABC type transporter with a bacteriocin secretion accessory protein (Figure 5C and Supplementary Table 6). In the cassette, there are also a Blp family class II bacteriocin, an MFS transporter and a PTS sugar transporter subunit IIA.

BAGEL4 did not find bacteriocin cassette in antimicrobial *W. paramesenteroides* W31. However, when comparing the bacteriocin sequences from DmW_118 and DmW_118119 to the proteome of W31, a similar 45-aa leucocin A/sakacin P class II bacteriocin with 23-aa double glycine signal peptide was found but annotated as hypothetical protein (80.95% identical, excluding the signal peptide, Figure 5B). When this putative W31-bacteriocin was compared with other *W. paramesenteroides* proteomes, identical or nearly identical proteins were found from all of them, including the idli strains B1 and C8, which have not shown any antimicrobial activity. All these putative bacteriocins shared a YWGNGV consensus motif, resembling the YGNGV “pediocin box” motif of class IIa bacteriocins. The 297-bp gene immediately downstream of the leucocin A/sakacin P family bacteriocin encoding gene (Figure 5C) was also found from other *Weissella* genomes and shared 59% identity with that from DmW_118, DmW_118119, and W31. The location and the size of this gene suggested its role as a bacteriocin immunity protein. Other bacteriocin related genes, e.g., those encoding bacteriocin transporters, were not found from the rest 39 strains.

### Antibiotic resistance and virulence

The genomes of all 42 strains were screened for antibiotic resistances in RGI online tool. All showed “Strict” hits to *vanT* gene from glycopeptide resistance gene cluster *vanG*. Three SJ strains also returned “Perfect” hits on *mefF* major facilitator superfamily (MFS) antibiotic efflux pump, which causes macrolide antibiotic resistance, and “Strict” hits on *msrG msr*-type ABC-F protein, macrolide and streptogramin antibiotic resistance. DmW_118 and DmW_118119 had the Strict hits to *qacJ*, encoding small multidrug efflux pump (Supplementary Table 7). Since some *Weissella* spp. like *W. cibaria* and *W. confusa* have caused infections to human, we also screened the pathogenicity of all the 40 *W. paramesenteroides* strains with VirulenceFinder. But none of the genomes was found to carry genes associated with virulence.

## Discussion

In this study, we sequenced and assembled six genomes of *W. paramesenteroides* isolates. Together with the already available 36 genomes of this species, we compared all 42 genomes and decipher the functional potential of these *Weissella* strains.

Genome comparison indicated that 40 strains of *W. paramesenteroides* are a compact group of bacteria, with values of sequence similarity much higher (ANI > 97.7%) than the threshold required for species demarcation, although their pangenome remained open. Two strains, DmW118 and DmW118119, are outliers of *W. paramesenteroides* (ANI 84.4%) and according to our analysis should be considered as *W. hellenica*.

The pangenome of *W. paramesenteroides* remained open. However, through sequence alignment of the core genome, three major clades of the strains were revealed (Figure 2B). The *W. paramesenteroides* DmW strains isolated from the gut of common fruit flies (*Drosophila melanogaster*) (Rudman et al., 2019) compose predominantly in two clades: one clade included also strain STCH-BD1 [isolated from ensiled sorghum (*Sorghum bicolor*)], and the other DmW-clade composes also 5 of our newly sequenced strains B1, W31 (isolated from idli batter), SJ21-9, SJ27-4, and SJ27-5 (isolated from air dried onion powders). The DRD strains isolated from artisanal Greek cheeses such as Feta and Kefalograviera dominated the 3rd major clade and shared a common ancestor with strains ATCC 33313, MGYG-HGUT-01340, FDARGOS_414, and Wpk4. These observations suggest the persistence of *W. paramesenteroides* species in a variety of fermented dairy products and vegetative environment such as vegetable preservation, grain fermentation/farming.

The majority of functional core genome of *W. paramesenteroides* in our study here was found to be associated with amino acid and nucleotide metabolism and transport (Figure 3 and Supplementary Table 2), which indicate that this species could utilize various amino acids and nucleotides and have a potential in heterofermentation. Although the wheat-related grains are generally low in lysine, threonine and sulfur-containing amino acids (Jiang et al., 2008) whilst legumes are often limiting in methionine and tryptophan (Semba et al., 2021), combination of various vegetative sources in fermentation in practice allows *W. paramesenteroides* make full use of its metabolic pathways. Secondary metabolites often determine the overall flavor and organoleptic properties in fermented foods, particularly in alcoholic beverages (Stewart, 2017). Moreover, extracellular small molecular metabolites by lactic acid bacteria indeed exhibit anti-inflammatory activities against gastrointestinal pathogens (Kostelac et al., 2021). Taking the good number of transmembrane transporters harbored in *W. paramesenteroides* core genome into consideration (Supplementary Table 2), these strains could also possess similar anti-inflammatory properties. Further and detailed analysis on the compositions of *W. paramesenteroides* extracellular secondary metabolites and vitamins could convict their role to a beneficial effect on human health through food fermentation.

Production of bacteriocins is often a plasmid-mediated trait. As revealed in this study that *W. paramesenteroides* strains rarely carry plasmids, it is not surprising that only a few bacteriocins have been reported from *W. paramesenteroides*, such as the well-characterized weissellin A, unclassified bacteriocin BacJ1 and a nameless peptide from the strain DFR-8 (Pal and Ramana, 2010; Papagianni and Papamichael, 2011; Chakchouk-Mtibaa et al., 2014). In our study here, the possible bacteriocin cassettes were only found from the chromosomes of strains DmW_118 and DmW_118119, which should be identified as *W. hellenica*. All 40 true *W. paramesenteroides* genomes carried a gene encoding protein resembling a class IIa bacteriocin. However, as the same gene was found from both antimicrobial (W31) and non-antimicrobial (B1, C8) strains, its function is questionable. Experimental evidence of cloning and expressing the gene would be needed to confirm its bacteriocinogenicity. Although the plasmid of the antimicrobial strain W31 did not carry any obvious bacteriocin genes, we cannot exclude the possibility that some plasmid-borne hypothetical genes would still encode bacteriocins.

Like many lactic acid bacteria, *W. paramesenteroides* strains also possess intrinsic vancomycin resistance by harboring *vanT* gene in their genomes (Supplementary Table 7), which make their cell wall poorly affinitive to vancomycin. As the last resort for treating Gram-positive bacterial infections, vancomycin inhibits the cross-linking of the peptidoglycan matrix by binding to the D-Ala-D-Ala terminal of the growing peptide chain during cell wall synthesis (Schäfer et al., 1996). Yet in vancomycin resistant Gram-positive bacteria, the D-Ala-D-Ala terminus is often replaced with D-Ala-D-Lac or D-Ala-D-Ser to which vancomycin has low affinity. The *vanT* gene found in the VanG resistance cluster encodes a membrane-bound serine racemase (Depardieu et al., 2003). The VanT racemase catalyzes the conversion of L-Ser to D-Ser in the production of D-Ala-D-Ser terminator (Meziane-Cherif et al., 2015). Although *Weissella* are intrinsically resistant to vancomycin (Ahmed et al., 2022), the resistance is most likely not easily transferred to pathogenic microbes. A very recent study by Fhoula et al. (2022) showed that although *W. paramesenteroides* strain could receive antibiotic resistance genes from *Bacillus* strains at very low frequency of 10^–9^ transconjugant per recipient, *Weissella* strains were not able to transfer their antibiotic resistance genes to *Enterococcus* strains.

Biogenic amines accumulated by the microorganisms in fermented foods can cause adverse effects to human (Alvarez and Moreno-Arribas, 2014). Many *Weissella* spp. such as *W. cibaria* and *W. confusa* have caught attention of their conversion of amino acids to biogenic amines (Jeong and Lee, 2015). Nevertheless, the production of biogenic amines is rather a strain-dependent trait (Jeong and Lee, 2015; Takebe et al., 2016), and often requires high level of protein content in the fermented food matrix such as in fish products (Ruiz-Capillas and Herrero, 2019). All the 40 *W. paramesenteroides* strains in our study did not carry decarboxylase genes required for the conversion of amino acids (histidine, lysine, ornithine, and tyrosine) to biogenic amines (histamine, cadaverine, putrescine, and tyramine, respectively). Therefore, it is unlikely that these *W. paramensenteroides* strains would cause the accumulation of biogenic amines through food fermentation.

The evidence in our study supports the use of *W. paramesenteroides* from environmental sources as starter cultures in food fermentation, yet a more detailed *in silico* examination of their absence of transposons and phenotypic and genetic virulence determinants will ensure their safe status in the food application.

## Data availability statement

The datasets presented in this study can be found in online repositories. The names of the repository/repositories and accession number(s) can be found in the article/Supplementary material.

## Author contributions

TS, RK, and PS conceptualized the study. XW and RK designed the experiments. SA and VH carried out the wet lab experiments, including growing cells, and DNA extraction. TT and JB supervised the wet lab experiments. LP sequenced the genomes and performed the genome assembly. RK performed the bioinformatics for genome assembly. XW performed all sequence and data analyses and wrote the manuscript with contributions from TT, JB, LP, PS, RK, SA, TS, and VH. All authors reviewed the article and approved the submitted version.

## Footnotes

1 https://github.com/sanger-pathogens/Roary/tree/master/contrib/roary_plots (accessed 6 July, 2022).

2
http://bagel4.molgenrug.nl/

3
https://cge.food.dtu.dk/services/VirulenceFinder/

4
https://card.mcmaster.ca/analyze/rgi

5
http://eggnog-mapper.embl.de/

6
https://www.genome.jp/kegg/mapper/reconstruct.html
