# Supplementary material for: Comparative genomics of 40 Weissella paramesenteroides strains
Source: Front Microbiol. 2023 Mar 31;14:1128028. doi: 10.3389/fmicb.2023.1128028 (PMC10102382; doi:10.3389/fmicb.2023.1128028)
Supplement: Supplementary file 2 [file Image_1.pdf]

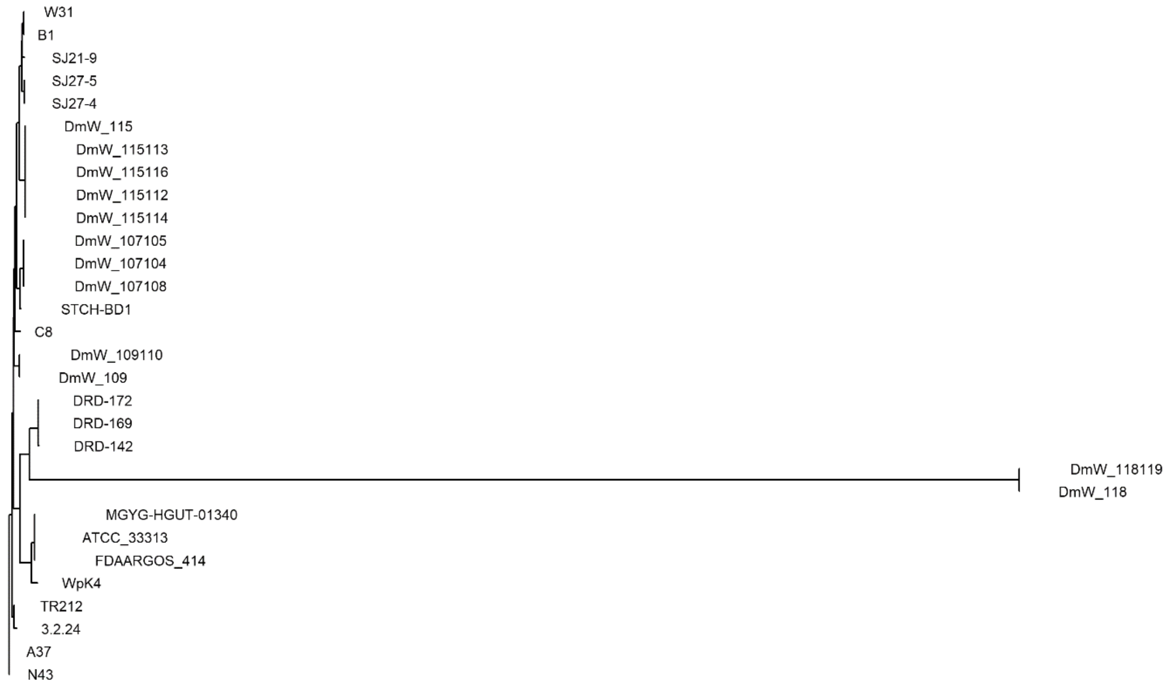

Supplementary Figure 1. Unrooted phylogenetic tree of all 42 publicly available *W. paramesenteroides* genomes
